# Fibrin Hydrogels for Endothelialized Liver Tissue Engineering with a Predesigned Vascular Network

**DOI:** 10.3390/polym10101048

**Published:** 2018-09-20

**Authors:** Xiaohong Wang, Chang Liu

**Affiliations:** 1 Department of Tissue Engineering, Center of 3D Printing & Organ Manufacturing, School of Fundamental Sciences, China Medical University (CMU), No. 77 Puhe Road, Shenyang North New Area, Shenyang 110122, China; 2 Center of Organ Manufacturing, Department of Mechanical Engineering, Tsinghua University, Beijing 100084, China; 3 Tianjin Mifang Science & Technology Ltd., Wuqing 301701, China; fluent_benjamin@yeah.net

**Keywords:** fibrin hydrogel, artificial livers, rotational combined mold, branched vascular network, adipose-derived stem cells (ADSCs)

## Abstract

The design and manufacture of a branched vascular network is essential for bioartificial organ implantation, which provides nutrients and removes metabolites for multi-cellular tissues. In the present study, we present a technology to manufacture endothelialized liver tissues using a fibrin hydrogel and a rotational combined mold. Both hepatocytes and adipose-derived stem cells (ADSCs) encapsulated in a fibrin hydrogel were assembled into a spindle construct with a predesigned multi-branched vascular network. An external overcoat of poly(dl-lactic-*co*-glycolic acid) was used to increase the mechanical properties of the construct as well as to act as an impervious and isolating membrane around the construct. Cell survivability reached 100% in the construct after 6 days of in vitro culture. ADSCs in the spindle construct were engaged into endothelial cells/tissues using a cocktail growth factor engagement approach. Mechanical property comparison and permeability evaluation tests all indicated that this was a viable complex organ containing more than two heterogeneous tissue types and a functional vascular network. It is, therefore, the first time an implantable bioartificial liver, i.e., endothelialized liver tissue, along with a hierarchical vascular network, has been created.

## 1. Introduction

There are about 78 organs in a human body [1]. Each of the organs consists of multiple heterogeneous tissues. Visceral organs, such as the liver, heart, kidney and lung, are essential for human survival. In particular, the liver is the largest digestive organ in the human body. In contrast to the structural or sensorial (or sensory) organs, such as bone, cartilage and skin, the liver carries out more than hundreds of metabolic functions, such as synthesis, transformation and excretion. Historically, acute liver failure remains a disease with very high mortality [2]. The severely limited donor supply, the lifelong immune exclusion reaction and the extremely high transplantation cost have stimulated continuing efforts to manufacture implantable bioartificial livers with a predesigned vascular network [3,4,5,6,7,8].

Over the last several decades, the manufacture of an implantable predesigned vascular network has been a long-pursued goal for many researchers in some research fields, such as cell therapies, tissue engineering and regenerative medicine. Numerous approaches have been exploited using both isolated cells and other biomaterials [9,10,11,12]. Some manufacturing technologies, such as photolithography, microfluidic molding and three-dimensional (3D) printing, have been adapted or employed [13,14,15,16]. The early work on vascular network generation relies mainly on the stacking of two-dimensional (2D) cell-laden hydrogels or coculturing of endothelial cells (ECs) with other types of cells in a soft hydrogel. For example, Kim et al. made an endothelial cell layer for a rolling blood vessel formation using a stereolithography technique [17]. Levenberg et al. built a vascularized skeletal muscle construct from coculturing myoblasts, ECs and fibroblasts [18]. Chen et al. employed a photopolymerizable gelatin methacrylate hydrogel to support blood-derived endothelial colony-forming cells and bone marrow-derived mesenchymal stem cells [19]. Zheng et al. fabricated a tubular endothelialized microchannel using a collagen matrix [20]. Golden et al. used a sacrificial micromold to create microfluidic meshes in collagen and fibrin hydrogels [21,22]. Miller et al. printed carbohydrate glass to form latticed vascular-like networks [23]. Wu et al. constructed fugitive ink networks within a photocrosslinkable gel [24]. It is recognized that a predesigned hierarchical (i.e., multi-branched) vascular network is essential for bioartificial organ manufacturing to ensure long-term survival of the implanted grafts [25,26,27,28,29].

**Figure 1:**
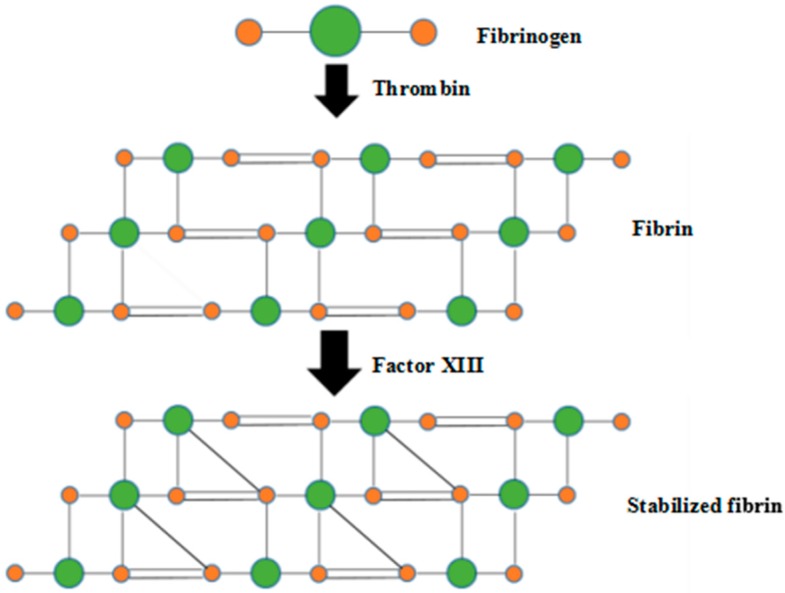
Fibrin formation and stabilization through thrombin and activated factor XIII.

Fibrin is a non-globular fibrous protein involved in the clotting of blood (Figure 1) [30]. It is formed by the polymerization of fibrinogen acted by protease thrombin. The polymerized fibrin, together with platelets, forms a hemostatic plug or clot over a wound site to protect the wound tissues from infection. Fibrin has been widely used in biomedical fields as nanoparticles, porous scaffolds, cell sheets and 3D-printed constructs [31]. The excellent biocompatibility and 3D processable capabilities of fibrin hydrogel have made it a suitable candidate for various bioartificial organ manufacturing.

In our previous studies, we have developed a series of organ manufacturing strategies, such as the combined multi-nozzle rapid prototyping (i.e., 3D bioprinting) technologies, cocktail stem cell engagement protocols and additive combined mold systems (ACMS) [32,33,34,35,36,37,38]. Among these strategies, ACMS is a semi-automated organ manufacturing technology that allows precise and repeatable placement of multiple cell types, extracellular matrices (ECMs) and/or other important biological factors (i.e., bioactive agents, molecules or components) in a predesigned manner.

Here we report a different approach for producing an implantable endothelialized liver tissue with hepatocytes and adipose-derived stem cells (ADSCs) in a fibrin hydrogel with an outer layer of supportive poly(dl-lactic-*co*-glycolic acid) (PLGA) by the use of an ACMS technology. Heterogeneous cell fates are controlled by using the predefined 3D printed molds. Multiple cell types behave sensitively in the constructs and the implantable endothelialized liver tissue performs biological functions over a 9-day in vitro culture. Compared with other existing technologies, the ACMS technology is a relatively simple manufacture approach which can better replicate the 3D cell survival environments of a complex visceral organ for producing the multi-branched vascular network under benign conditions. This is a critical step or a huge milestone in bioartificial organ manufacturing areas.

## 2. Materials and Methods

### 2.1. Materials

Fibrinogen was purchased from Sigma (Sigma, St. Louis, MO, USA). A 1% (*w*/*v*) fibrinogen solution was prepared by dissolving bulk fibrinogen in fetal bovine serum (FBS) (Sijiqing, Beijing, China) free Dulbecco’s modified Eagle’s medium (DMEM) (Gibco, Vienna, VA, USA). Bovine thrombin (Gibco, Santa Cruz Biotechnology, Santa Cruz, CA, USA) was dissolved in deionized water to give a final concentration of 20 IU/mL. PLGA (LA/GA = 70:30, *M*_w_ = 100,000 Da, Institute of Medical Devices of Shandong Province, Jinan, China) was dissolved in tetraglycol (Sigma, St. Louis, MO, USA) to form a 15% (*w*/*v*) solution. Dimethyl sulfoxide (DMSO) and other chemicals were all of analytic grade and purchased from Beijing Organic Chemical Plant, Beijing, China.

### 2.2. Hepatocytes and ADSCs

Hepatocytes were isolated from the liver of an 8-week-old Sprague-Dawley male rat (150 g from the Laboratory Animals Center, Medical Department of Beijing University, Beijing, China) using an in situ collagenase perfusion method and cultured in DMEM containing 10% FBS at an incubator at 37 °C and 5% CO_2_ before assembling. ADSCs were isolated from subcutaneous adipose tissues of the same rat.

### 2.3. Construction of a Spindle Endothelialized Liver Tissue

**Figure 2:**
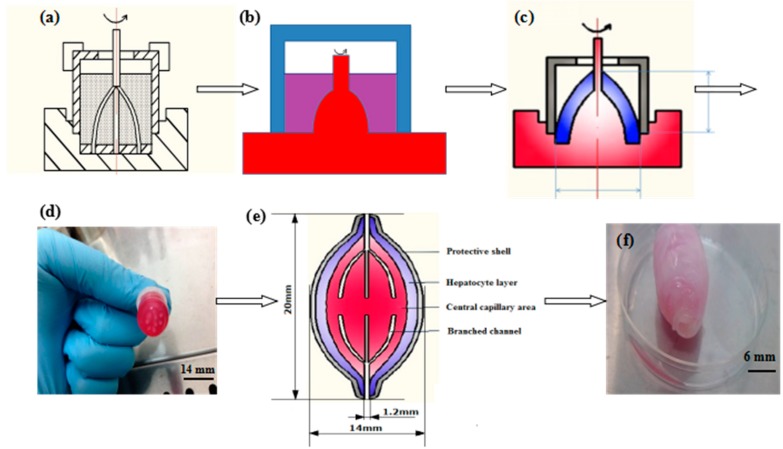
Configuration and manufacturing process of a spindle vascularized liver tissue: (**a**) schematic illustration of the cell-laden fibrinogen dopant distributed in between the rotational combined mold system before rotation; (**b**) the cell-laden fibrinogen hydrogel (red) covered the inner branched mold and stabilized using thrombin solution (purple); (**c**) the first layer of adipose-derived stem cell (ADSC)-laden fibrin hydrogel (blue); (**d**) a semi-spindle construct containing two layers of ADSC and hepatocyte-laden fibrin hydrogels among the rotational combined mold system with microholes in the bottom mold; (**e**) a computer aided design model with the inner branched mold, ADSC-laden fibrin hydrogel, hepatocyte-laden fibrin hydrogel, and poly(dl-lactic-*co*-glycolic acid) overcoat; (**f**) an implantable spindle endothelialized liver tissue made of two semi-spindle constructs containing two predesigned multi-branched vascular networks derived from a ADSC-laden fibrin hydrogel, a living hepatic tissue, and a poly(dl-lactic-*co*-glycolic acid) overcoat.

The ACMS technology was established to assemble different cell-laden fibrin hydrogels layer-by-layer into a large construct with a branched vascular network inside and a PLGA overcoat (Figure 2) [36]. It is a unique additive combined rotational mold system. Briefly, the ACMS consists of an internal 7-branched polyethene mold derived from a center pole, a 3D-printed polytetrafluoroethlyene basement mold with 7 blind holes on one side and a series of 3D-printed polytetrafluoroethlyene external molds with different diameters. ADSCs and hepatocytes either mixed with the fibrinogen solution separately or together. During the fabrication stage, a mixture of ADSCs and fibrinogen solution 1% (*w*/*v*) with a cell density of 1 × 10^6^ or 3 × 10^6^ cells/mL was injected into the lumen among the internal branched mold, basement mold and first external mold before the hepatocyte-laden fibrinogen layer (hepatocyte density in the fibrinogen solution was 5 × 10^6^ cells/mL). ADSC density in the middle connection layer was 3 × 10^6^ cells/mL. In some spindle constructs a mixture of ADSCs (1 × 10^6^ cells/mL) and hepatocytes (5 × 10^6^ cells/mL) was used to substitute the pure hepatocyte layer. Two semi-spindle constructs with a precise control over the internal/external architecture harboring both hepatocytes and ADSCs were connected back to back to construct a spindle construct (Figure 2). Noncellular, non-fibrinogen hydrogel and non-PLGA groups were used as controls.

### 2.4. Cell Viability Tests

An acridine orange (AO)/propidium iodide (PI) staining method was used to test the live/dead cell states. AO can only pass through living cell membrane and has different binding capacity with DNA and RNA. It is a living cell fluorescent dye with an excitation wavelength of 492 nm and emission wavelength of 530/640 nm. It emits fluorescence in different colors (green fluorescence with DNA and orange or orange-red with RNA). PI is a dead cell fluorescent dye with an excitation wavelength of 535 nm and emission wavelength of 615 nm. It cannot pass through living cell membrane, but can pass through broken cell membrane. Thus, an AO/PI staining technique was used to test the live/dead cell states. Simply, a work solution was obtained by mixing 0.01 mL of AO solution (0.3 mg/mL, Sigma, St. Louis, MO, USA) and 1 mL of PI solution (0.5 mg/mL, Sigma, St. Louis, MO, USA) and diluting 10 times before it was filtered with a 0.22 μm membrane. The liver lobe precursors were washed with PBS three times before they were stained with the AO/PI work solution at an incubator at 37 °C for 10 min. After being washed with PBS for another three times, the samples were observed under a laser scanning confocal microscopy (LSCM, Zeiss, Jena, Germany, LSM710, AO 488 nm, PI 546 nm). The numbers of live and dead cells were counted using NIH ImageJ program. Three time points (i.e., 3, 6, and 9 days) were chosen during the in vitro cultures. Each group was tested three times in parallel wells.

In parallel, a 3-[4,5-dimethylthiazol-2-yl]-2, 5-diphenyl tetrasodium bromide (MTT) method was used to evaluate the cell proliferation rate. After culturing for different periods (i.e., 0, 3, 5, 7, 9 days), a part of the precursors was cut into small pieces (1 × 1 × 1 mm^3^), weighed, placed into a 96-well plate with 100 μL of DMEM, and incubated with 20 μL of a 5 mg/mL MTT solution at 37 °C for 4 h. Then, 150 μL of DMSO was added to each well and the culture plate was shaken at a low speed for 10 min to fully dissolve the crystals. The optical density (*OD*) of each well was measured at 490 nm wavelength using a Bio-Rad 680 reader (OEM Systems Co., Ltd., Tokyo, Japan). During the MTT measuring process, the weight of the samples was recorded as *W_ij_* (*i* = 0, 1, 2, 3, 4 days, *j* = the number of samples of 1, 2, 3, ·····), and the corresponding absorbance value *OD_ij_* was obtained. Cell proliferation rate, *R_i_*, was calculated according to the following Equation (1) or (2). *OD* of the fibrin hydrogel sample with no cells inside was set as zero.

(1) $$R_{i}=\frac{\sum_{j=1}^{3}OD_{ij}-\sum_{j=1}^{3}OD_{ij}\times (\sum_{j=1}^{3}W_{ij}-\sum_{j=1}^{3}W_{0j})/\sum_{j=1}^{3}W_{ij}}{\sum_{j=1}^{3}OD_{0j}}-1$$

(2) $$R_{i}=\frac{\sum_{j=1}^{3}\bar{OD_{ij}}}{\sum_{j=1}^{3}W_{ij}}/\frac{\sum_{j=1}^{3}\bar{OD_{0j}}}{\sum_{j=1}^{3}W_{0j}}-1$$

where Equation (2) is an extension of Equation (1). *R_i_* represents *i* days of in vitro culture; the cell proliferation rate is the value that was obtained from the ratio of the *OD* value at day *i* to the original *OD* at day 0 after subtracting 1. The cell number is proportional to the *OD* value. Each group was tested in triplicate in parallel wells.

### 2.5. In Vitro Engagement and Characterization of the Cells

To study the inducement effects of the ADSCs to ECs in the spindle constructs, two groups of independent experiments with low and high ADSC densities of 1 × 10^6^ and 3 × 10^6^ cells/mL were set. ADSCs in each group were engaged towards ECs for 0, 5, and 10 days with a cocktail addition of 50 ng/mL vascular EC growth factor (VEGF), 3 ng/mL transforming growth factor *β*_1_ (TGF-*β*_1_) and 10 ng/mL basic fibroblast growth factor (b-FGF) in the culture medium after the fabrication stage. There were 6 samples in each group [34].

After the engagement stages, the 3D constructs were cut into 2 × 2 × 1 mm^3^ pieces and washed three times with phosphate-buffered saline (PBS) (pH 7.2). Some of the pieces were firstly incubated with 10% (*v*/*v*) FBS containing PBS for 30 min at room temperature. Then the samples were incubated with a primary antibody rabbit anti-FacVIII-rag at 37 °C for over 12 h before being incubated with a secondary antibody rabbit anti-human IgG (H+L) (tetramethylrhodamineisothiocyanate, TRITC) for 3 h. Finally, the samples were incubated with a 4′,6-diamidino-2-phenylindole (DAPI) solution and washed 3 times with PBS and observed with the above mentioned LSCM. The excitation and emission wavelengths for DAPI staining were 340 and 488 nm. Red fluorescence means that the cells expressed the endothelial marker FacVIII, while purplish blue means that the nuclei were stained. Cells encapsulated in the fibrin hydrogel in the 3D constructs without engagement were used as negative controls.

Some of the pieces were fixed in a 2.5% (*v*/*v*) glutaraldehyde solution at 4 °C for 2 h. The samples were firstly washed three times with PBS, before being dehydrated following the concentration gradient: ethanol in PBS 50%, 70%, 80%, 90%; 100% ethanol; ethanol in *tert*-butyl alcohol 50%; 100% *tert*-butyl alcohol. Then the samples were put in *tert*-butyl alcohol at −20 °C. After dehydration, the samples were freeze-dried and examined using a scanning electron microscopy (SEM), (Hitachi S-450, Hitachi, Tokyo, Japan). Some of the pieces were fixed in a 4% (*v*/*v*) paraformaldehyde solution for 4 h. Then the samples were washed three times with PBS, frozen sliced (12 μm in thickness), and stained with hematoxylin and eosin (HE). Each sample was tested three times.

To study the growth factor effects of hepatocytes on ADSCs, a mixture of ADSCs (1 × 10^6^ cells/mL) and hepatocytes (5 × 10^6^ cells/mL) in the fibrinogen solution was used as the second cell-laden layer for some of the spindle constructs. Some single ADSC-laden fibrin layer spindle constructs with a density of 3 × 10^6^ cells/mL of ADSCs and two cell (i.e., ADSC and hepatocyte)-laden fibrin layer spindle constructs with a density of 5 × 10^6^ cells/mL of hepatocytes and 3 × 10^6^ cells/mL of ADSCs were used as controls. The single ADSC-laden fibrin layer in the spindle constructs was also engaged with endothelial cell growth factor containing culture medium. After 0, 3 and 6 days of in vitro engagement, the samples were characterized using fluorescence staining. Some of the hepatocytes were stained with 5- or 6-(*N*-Succinimidyloxycarbonyl)-3′,6′-*O*,*O*′-diacetylfluorescein (CFSE) by adding 40 μL CFSE (1 μg/mL) into 1 mL of the hepatocyte suspension before being assembled and characterized using LSCM after 9 days in vitro culture. The excitation/emission wavelength for CFSE staining was 490 nm/530 nm. Each sample was tested three times.

Some samples were cultured in DMEM with 10% FBS (*v*/*v*). Metabolic activities of the hepatocytes were evaluated by measuring the secreta contents in the culture media after in vitro culturing for 1, 2, 3 and 4 days. The culture media of the spindle constructs were collected at different periods and stored at −20 °C for assay. After being centrifuged at 3000 rpm/min for 5 min, the media were analyzed using a Hitachi 7600 series automatic biochemical analyzer (Hitachi, Tokyo, Japan) for different biochemical parameters, such as glutamate-oxaloacetate transaminase (GOT), creatinine (Cr), urea (Ur), triglyceride (TG), total protein (TP) and albumin (ALB). The fresh culture medium and the media used for no hepatocyte constructs were used as negative controls. Meanwhile the rat serum was used as a positive control.

Some samples were fixed with 4% (*v*/*v*) paraformaldehyde for 4 h, cut into longitudinal or transverse sections of 20 μm thickness, washed 3 times with PBS, and then dehydrated using an ascending series of ethanol. After dehydration, the samples were freeze-dried and examined using a FEI Quanta 200 scanning electron microscopy (SEM) (Hitachi S-450, Hitachi, Tokyo, Japan), with some sections being stained with hematoxylin and eosin (HE). Each sample was tested three times.

### 2.6. Permeability of the Spindle Vascularized Liver Tissues

The permeability of the spindle constructs was tested by clamping the samples perpendicularly on an iron frame. A piece of accurate pH test paper was put under each sample. After HCI solution (0.1 M) was injected into the spindle construct through the inlet at a constant rate, the time was counted using a clock until the pH test paper had changed to red. Three groups of samples were tested. In group A, the spindle constructs were composed of ADSC and hepatocyte two cell layers and the ADSCs were engaged to ECs for 10 days. In group B, the spindle constructs were composed of ADSC and hepatocyte two cell layers with no growth factor engagement. In group C, the spindle constructs were composed of two fibrin hydrogel layers without cells.

### 2.7. Mechanical Strength Measurement

Axial tensilities of the spindle endothelialized liver tissues after 5 days of growth factor engagement are measured using a universal mechanical test machine (2000kgf PT-1080, DongGuan DongRi Instument, Co., Ltd., DongGuan, China). The drawing speed was set to 1 mm/min. Radial compliance of the samples was measured using a similar method as described previously according to ISO7198 [37,39]. These samples were set as the “A” group. The samples that had no cells inside were used as the control “B” group.

### 2.8. Statistical Analysis

Sample values were expressed as means ± standard deviation (SD). Unpaired Student’s *t*-test was used to compare the difference between the experimental groups containing different components. Statistical Product and Service Solutions (SPSS) version 13.0 statistic software (Chicago, IL, USA) was used to analyze the values. *p*-values < 0.05 were considered significant.

## 3. Results

### 3.1. Generation of the Spindle Cell-Laden Constructs

The semi-spindle constructs were formed when the ACMS was rotated at a specific speed (30 r/min) [36]. During the combined mold rotation stage, the cell-laden fibrinogen solution moved around the internal branched mold according to the Weissenberg effect of non-Newtonian fluid. After the fibrinogen molecules were polymerized with the thrombin solution, cells (i.e., ADSCs and hepatocytes) were immobilized in different layers of the fibrin hydrogels and the cell-laden fibrin hydrogels took the shape of membranes (Figure 2c). The rotation speed had a significant effect on the semi-spindle cell-laden membrane structures. At the beginning (~10 r/min) most of the cell-laden hydrogels were deposited on the bottom of the mold lumen. When the rotation speed reached 20 r/min, the climbing pole phenomenon of the cell-laden fibrin hydrogels became more evident in the semi-spindle constructs. At 30 r/min, most of the internal branched molds were covered by the cell-laden fibrin hydrogels with a slim semi-spindle structure.

A spindle construct with two multi-branched vascular networks and two cell lines is shown in Figure 2. The vascular networks include one central artery-like channel, 7 branched arteriole-like channels, a middle capillary-like layer, 7 branched venule-like channels and a central vein-like channel. Each of the semi-spindle cell-laden construct contains a multi-branched vascular network (Figure 2a,e). When the two semi-spindle constructs were connected with a thin layer of ADSC-laden fibrin hydrogel, a complete vascular network (mimicking the natural arteries, arteriolae, capillaries, venulae and veins in a visceral organ) was constructed (Figure 2c,e). Tight connections between the two embodied semi-spindle constructs were achieved using a layer of middle ADSC-laden fibrin hydrogel, with a continuous PLGA overcoat extending from the two semi-spindle constructs. The 7 branched channels are not directly connected between the two halves in case of the “short circuit”. Thus, the vascular network includes one inlet (mimicking the artery), 7 branched arteriole-like channels, a capillary-like middle layer in between the two semi-spindle constructs, 7 branched venule-like channels and an outlet (mimicking the vein). The middle capillary-like layer can protect the fluid from “short circuit”, i.e., going directly from the arteriole-like channels to the venule-like channels, mimicking the real capillary structures in the liver.

### 3.2. Cell Viability in the Spindle Construct

**Figure 3:**
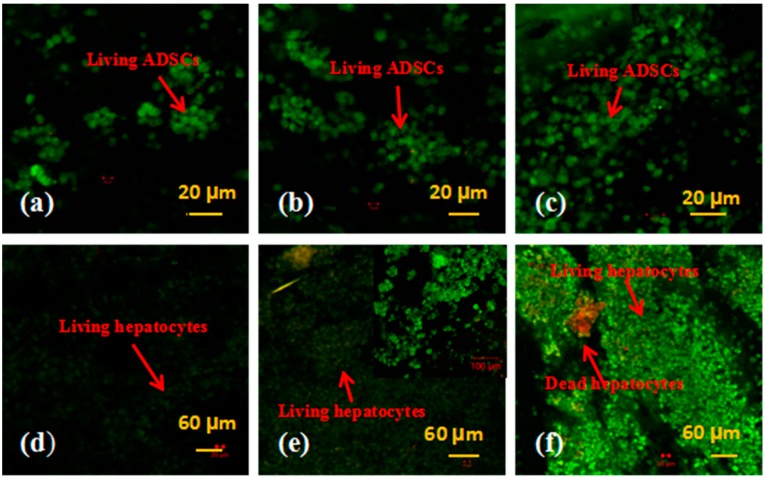
Live/Dead states of the adipose-derived stem cells (ADSCs) and hepatocytes in the spindle construct after different in vitro culture periods: (**a**) ADSCs 3 days; (**b**) ADSCs 6 days; (**c**) ADSCs 9 days; (**d**) hepatocytes 3 days; (**e**) hepatocytes 6 days, a magnified image on the upper right corner to show the living cells more clearly; (**f**) hepatocytes 9 days. Red color stands for dead cells, while green color stands for living cells.

Live/dead cells in the spindle construct were firstly detected using a LSCM after AO-PI staining. Dead cells appeared red for absorbing the PI dye, while living cells presented green with the AO. Figure 3 represents the detection results of the ADSCs and hepatocytes encapsulated in the fibrin membranes after 3, 6, and 9 days of in vitro culture. Before the fabrication stage, both the ADSCs and hepatocytes were distributed in the fibrinogen solution evenly. After being assembled, cells in the fibrin membranes were reunited as aggregates (Figure 3a,b). Large cell aggregates were prominent in the 9-day samples. During the 9 days in vitro culture, all the ADSCs were stained green, suggesting a very high level of stem cell survival capability. Meanwhile, a few red colored hepatocytes were dead. Hepatocyte viability was more than 98%. These results indicate that the cell assembling procedures, the rotational combined molding, PLGA coating and organic solvent extraction processes have no significant effects on the cell viability.

**Figure 4:**
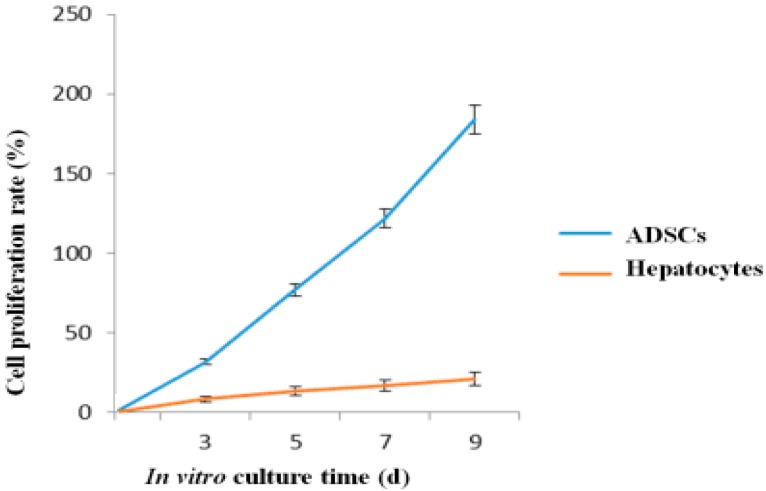
Adipose-derived stem cell and hepatocyte proliferation rates in the fibrin hydrogel within the spindle construct after different periods of in vitro cultures (*p* < 0.01).

In Figure 4, the proliferation rates are determined by MTT tests. Both the ADSCs and hepatocytes proliferated during the 9 days of in vitro culture. The proliferation rates of the two types of cells were not linear with the cell densities and culture times. In particular, the proliferation graph of the ADSCs increased very sharply compared with that of the hepatocytes. These results indicate that the proliferation capability of the ADSCs was much higher than that of the hepatocytes over the 9 days culture. A significant difference was found between the two groups containing ADSCs and hepatocytes (*p* < 0.01).

### 3.3. In Vitro Engagement Effects of ADSCs to ECs and Hepatocytes in the Spindle Constructs

**Figure 5:**
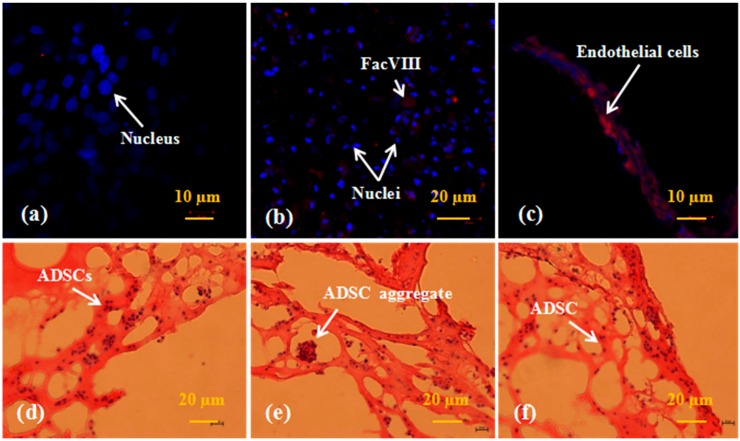
In vitro engagement effects of ADSCs (low cell density: 1 × 10^6^ cells/mL) to ECs at different culture periods: (**a**) immunofluorescence staining at the first day before engagement showing the purplish-blue ADSC nuclei with negative FacVIII marker (red); (**b**) immunofluorescence staining at the fifth day showing that the dark red FacVIII marker emerged with reduced blue purplish-marker; (**c**) immunofluorescence staining at the tenth day showing that the FacVIII were positive (bright red) with further reduced purplish-blue; (**d**) HE staining at the first day before engagement showing that the ADSCs encapsulated in the fibrin hydrogel randomly with a lot of micropores in the fibrin hydrogel; (**e)** HE staining at the fifth day showing that most of the ADSCs in the fibrin hydrogel were elongated around the channels, with some large cell aggregates and macropores; (**f**) HE staining at the tenth day, showing that a thin layer of elongated ADSCs on the surface of the cell-laden fibrin hydrogel.

**Figure 6:**
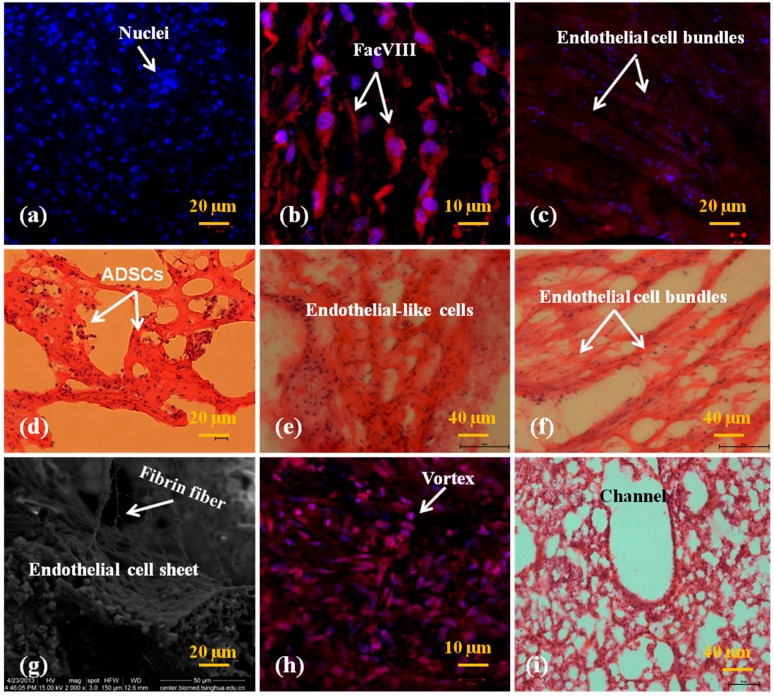
In vitro engagement effects of ADSCs (high cell density: 3 × 10^6^ cells/mL) to ECs at different times: (**a**) immunofluorescence staining at the first day before engagement showing that the nuclei of the ADSCs were purplish-blue with negative FacVIII marker; (**b**) immunofluorescence staining at the fifth day showing that the purplish-blue marker decreased while the dark red FacVIII marker emerged and the cells were elongated along a uniform direction; (**c**) immunofluorescence staining at the tenth day showing that the purplish-blue marker further decreased with a dense layer of FacVIII marked red cell clusters; (**d**) HE staining at the first day before engagement showing that the ADSCs encapsulated in the fibrin hydrogel randomly with a few small cell aggregates; (**e**) HE staining at the fifth day showing that most of the ADSCs in the fibrin hydrogel were elongated; (**f**) HE staining at the tenth day showing an elongated ADSC sheet on the surface of the cell-laden fibrin hydrogel; (**g**) a SEM image of the endothelial-like cell sheet along a branched channel at the tenth day in the spindle construct with some fibrin fibers; (**h**) immunofluorescence staining showing the endothelial-like cells in the joint middle cell-laden fibrin hydrogel layer with a high original cell density at the tenth day engagement; (**i**) a HE photo showing the cell-laden fibrin hydrogel at the end of a branched channel at the tenth day engagement.

As stated above, the two semi-spindle constructs were connected with a thin layer of ADSC-laden fibrin hydrogel. After ADSCs in the fibrin hydrogel were engaged to ECs, a vascular network was formed with go-through branched channels inside the spindle constructs. Figure 5 and Figure 6 show the immunofluorescence and HE staining results of the spindle constructs with low (1 × 10^6^ cells/mL) and high (3 × 10^6^ cells/mL) initial ADSC densities at different engagement stages. Along the two multi-branched vascular networks numerous honeycomb-like capillary-like structures formed (Figure 5d–f). Especially in the middle cell-laden fibrin connection layer, the capillary-like structures were more prominent, similar to those at the ends of the multi-branched arterioles and venules in a real native organ, such as the liver. The micropores are mainly a result of the high water content fibrin hydrogel, which benefits the capillary-like structure formation along the ADSC clusters. For the middle cell-laden fibrin connection layer, sufficient nutrient, gas and waste exchanges take place when the culture medium passes through the two separated multi-branched channel networks.

At the beginning of engagement (0 day), ADSCs in the spindle construct were negative with the FacVIII marker (Figure 5a and Figure 6a). There was no FacVIII secretion around the ADSCs. The nuclei of the ADSCs expressed purplish-blue color with the fluorescence of DAPI. All the cells were encapsulated in the fibrin membranes randomly with one or two (i.e., few or nearly no) large aggregates. Four days after engagement, some of the ADSCs in both the constructs with low and high concentrations of fibrin were elongated with positive endothelial marker FacVIII (red color in Figure 5b and Figure 6b). At the tenth day of engagement, all the ADSCs along the multi-branched channels expressed endothelial marker FacVIII and organized into large cell clusters (Figure 5c and Figure 6c,f,g). The purplish-blue colored nuclei were encapsulated by thick endothelial marker FacVIII in red color (Figure 5c and Figure 6c). Some of the cells were in proliferation stages with double-connected nuclei. Endothelial cell morphologies dominated both the low and high cell density samples. In particular, intima-like structure mimicking the inner layer of large blood vessels was much more prominent in the high cell density (i.e., low fibrin concentration) samples (Figure 6c,f,g). On the contrary, it was hard for the elongated ECs in the low cell density samples to form thick cell sheets. These results indicate that the ADSCs differentiated into ECs after the cocktail growth factor stimulation. The higher the ADSC density in the fibrin membrane was, the thicker the intima-like tissue formed.

Another interesting phenomenon is that the engagement effects in the middle cell-laden fibrin connection layer were obviously different from those around the multi-branched channels. In a HE staining picture, macropore structures were detected at the end of a branched channel (Figure 6i). There were no large cell clusters (or spheroids) in the middle cell-laden fibrin connection layer. However, plenty of small vortexes formed by endothelial cell-like organization were found in this layer. The sizes of the small vortexes in the middle cell-laden fibrin connection layer ranged from one to 20 µm (Figure 6h). Cells with red FacVIII marker extended along the small vortexes. The morphologies of the small vortexes were similar to the capillary structures in a natural liver.

**Figure 7:**
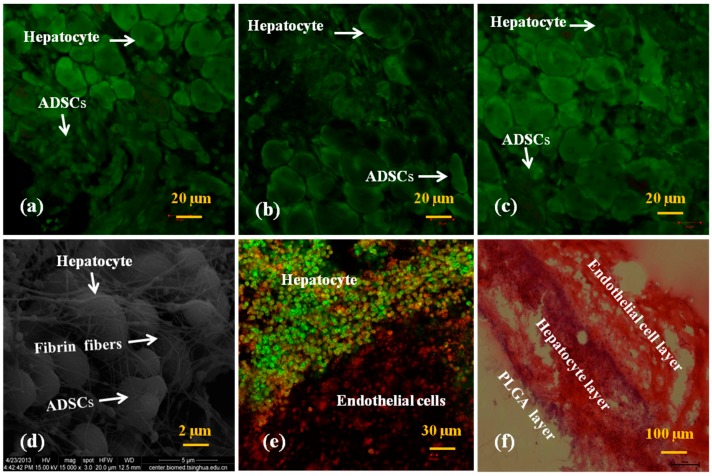
Laser scanning confocal microscopy (LSCM), scanning electron microscopic (SEM), and hematoxylin and eosin (HE) staining images of the mixtures of the adipose-derived stem cells (ADSCs) and hepatocytes after 0, 3, 6 and 9 days of in vitro culture: (**a**) 0 days, acridine orange/propidium iodide AO/PI live/dead staining for both the hepatocytes and ADSCs; (**b**) 3 days, AO/PI live/dead staining for both the hepatocytes and ADSCs; (**c**) 6 days, AO/PI live/dead staining for both the hepatocytes and ADSCs; (**d**) 9 days, a SEM image for both the hepatocytes and ADSCs; (**e**) 9 days, the interface of the hepatocyte and endothelial cell layers with CFSE staining for the hepatocytes, and FacVIII/DAPI staining for the endothelial cells (ECs); (**f**) 9 days, HE staining for both the hepatocytes and ECs.

In Figure 7a–c the mixed cells encapsulated in the fibrin membranes stained green, indicating that all the cells were alive after 6 days of in vitro culture. At the beginning of in vitro culture, two different cell sizes in the fibrin membranes were observed (Figure 7a). The average diameter of the hepatocytes was ~20 μm, which was much greater than that of the ADSCs, at ~5 μm. Three days after culture, the number of the small ADSCs was reduced. Some cells with diameters of between 10 and 5 μm appeared (Figure 7b). Six days later, the number of the small ADSCs was further decreased (Figure 7c). Nine days later, large sizes of hepatocyte aggregates were prominent (Figure 7d). Fibrin fibers were distinct in the SEM pictures (Figure 6g and Figure 7d). A clear interface of the hepatocyte/ADSC- and ADSC-loading fibrin membranes was demonstrated (Figure 7e,f). These results indicate that ADSCs in the first fibrin membrane differentiated into ECs with the growth factor engagement in the culture medium. ADSCs in the second fibrin membrane were affected by the growth factors either from the cocktail addition or the neighbour hepatocyte secreta. The influence of the growth factors from the neighbour hepatocytes is more obvious than that from the cocktail addition during the 9 days of in vitro culture. The morphological changes of the ADSCs mean that these stem cells could be induced into hepatocytes in the second fibrin membrane (Figure 7c).

### 3.4. Secretion Abilities of the Hepatocytes

**Figure 8:**
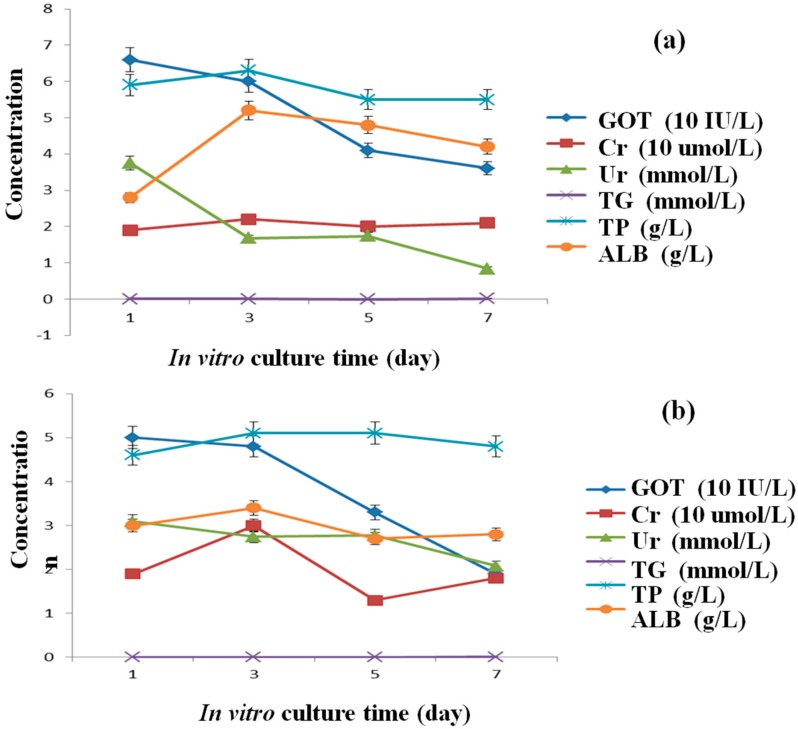
Secretion abilities of hepatocytes in the spindle construct after different in vitro culture periods: (**a**) ADSCs and hepatocytes mixed in the second cell-laden fibrin hydrogel layer; (**b**) hepatocytes alone in the second cell-laden fibrin hydrogel layer (*p* < 0.01).

Secretion capabilities of hepatocytes are important indexes for specific liver functions. Around the hepatocytes in the fibrin membrane, some small secreta (particles size < 1 μm in diameter) are evident (Figure 7c). These small particles could pass through the micro and macropores of the fibrin membrane and diffuse in the culture medium. Six secretion parameters of the hepatocytes in the ADSC/hepatocyte mixtures and pure hepatocyte groups are shown in Figure 8. In particular, GOT is a biochemical surrogate marker indicating hepatocyte necrosis and inflammation with acute hepatocellular injury [34,35].

From the curves in Figure 8, it is shown that both the GOT activities decreased during the in vitro culture periods, indicating that hepatocyte necrosis and inflammation decreased over time. Except TG maintained 0 in both the curves (Figure 8a,b), the values of Cr, TP and ALB increased to a certain degree before decreasing. The decreased level of Ur in curve b is not as high as the level of curve a. The decreased TG and Ur secretion functions may be due to the less supply of lipoid and sugar in the culture medium. The enhanced Cr, TP, and ALB synthetic activities of the hepatocytes, suggest that some liver-specific functions maintained in the spindle constructs during the 7 days of in vitro culture. These results confirm that hepatocytes were functional in the spindle constructs for more than one week. In the six secretion parameters, albumin is the most important index because the liver is the only place where albumin can be synthesized. After 7 days of in vitro culture, the average content of the ALB in the mixed cell group was 5.2 g/L, while in the control group was 3.4 g/L. These results indicate that it was a reasonable way to mix ADSCs and hepatocytes to prolong the hepatocyte activities.

### 3.5. Permeability of the Spindle Construct after ADSC Engagement

Table 1 shows the permeation times of the HCI solution in the three groups of spindle constructs. It took about 47.8, 57.1 and 61.4 s for the HCI solution passing through the spindle construct without cells, the spindle construct with double cell layers without growth factor engagement and the spindle construct with double cell layers and endothelial cell growth factor engagement for 10 days, respectively. The permeation time was greatly increased when the ADSCs were incorporated and engaged. A possible reason for this may be that it consumes more time for the HCI solution to go through the cell membranes inside the construct. After the engagement, ADSCs differentiated into ECs and arranged in order. Some intimate connections among the cells were built. It takes much more time for the HCI solution to cross over these special structures. The retention time was therefore prolonged. Significant difference was found between the three groups (*p* < 0.05). These results indicate that after being engaged by the growth factors, the fluid and cell interactions are more similar to those of a real vascular structure in the liver.

### 3.6. Mechanical Properties of the Spindle Vascularized Liver Tissues

**Figure 9:**
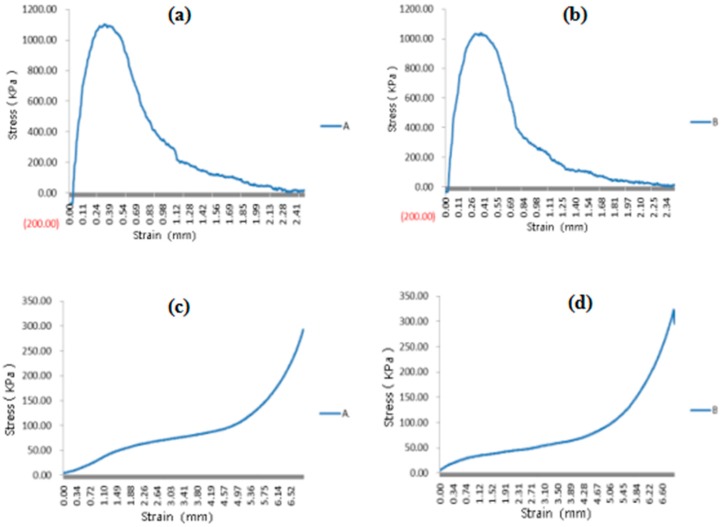
Mechanical properties of the spindle constructs: (**a**) tensilities (i.e., tensile strength) of the spindle endothelialized liver tissue after 5 days of growth factor engagement; (**b**) tensilities (i.e., tensile strength) of the spindle construct without cells inside immersed in the culture medium for the same period of (**a**); (**c**) compressive strength of the spindle endothelialized liver tissue after 5 days of growth factor engagement; (**d**) compressive strength of the spindle construct without cells inside immersed in the culture medium for the same period of (**c**).

The mechanical properties of the spindle constructs containing two heterogeneous cell types and no cells after 5 days in vitro culture are shown in Figure 9. The maximum tensile stress of the spindle constructs containing two heterogeneous cell types was 1038 KPa, while the control was 1104 KPa. Similar results were obtained for the compressive stresses. These results indicate that most of the mechanical properties of the spindle constructs were determined by the PLGA overcoat, not by the cells. The PLGA overcoat has played two roles. One of these is to increase the mechanical properties of the construct. The other is to act as an impervious and isolating membrane around the construct. During the in vitro culture and in vivo implantation stages, The PLGA overcoat also acts as a protective shell for the cell-laden fibrin hydrogels for more than one month before being biodegraded [36]. It was reported that the mechanical properties of the PLGA layers were gradually reduced after implantation. The elastic modulus was reduced from 85.2 to 69.3 MPa in 4 weeks, while the maximum stress was reduced from 1.31 to 1.02 MPa.

## 4. Discussion

Development of an implantable predesigned vascular network with multiple heterogeneous cell types is one of the key challenges for bioartificial liver manufacturing [39,40,41,42,43]. Polymeric hydrogels have played an essential role in various tissue engineering and organ manufacturing strategies [44,45,46,47,48,49,50,51,52,53,54]. In the present study, a spindle endothelialized liver tissue, comprised of an outer microporous PLGA layer, two internal cell-laden fibrin membranes, one middle capillary layer, together with two 7-branched vascular networks, is created using a brand-new ACMS technology. Each of the integrated vascular network consists of a central artery-like, seven-branched arteriole-like, seven-branched venule-like, a central vein-like and abundant capillary-like structures. This can be connected to the host vasculature directly to fulfill the required commitments of nutrient, gas and waste exchange for the incorporated cells. This spindle PLGA-coated endothelialized liver tissue construct has significant meanings for implantable bioartificial organ manufacturing and post clinical applications.

On the whole, the spindle endothelialized liver tissue construct serves as a spatiotemporal template for initial multi-cellular accommodation, subsequent heterogeneous tissue formation and later complex organ maturation. Beside the branched vascular networks, two outstanding parts in the 3D construct should be paid attention to. One of them is the PLGA overcoat. The PLGA overcoat provides stable mechanical support for the inner cell-laden fibrin membranes as well as the intricate vascular networks. The strong mechanical properties of the PLGA overcoat makes it possible for the spindle cell-laden construct to be connected to the host vasculature directly with anti-suture capabilities. The other part is the internal fibrin membranes which are made of the cell-laden fibrinogen hydrogels. The internal cell-laden fibrin membranes provide structural integrity to the heterogeneous cell types, lay down lumens to the branched vascular networks, allow full control of the ECM components, support nutrient/waste exchanges for the incorporated cells and determine the formation quality of the branching endothelia structures. With optimal fibrinogen concentrations before cell encapsulation, the internal cell-laden fibrin membranes could promote cell-cell interactions, new tissue generation and complex organ maturation to a great extent.

To construct the spindle construct containing a multi-branched vascular network, two half spindle constructs are first bonded together using the cell-laden fibrinogen hydrogel, and then sealed using a PLGA solution. Tight connections between the two embodied semi-spindle constructs are achieved using the cell-laden fibrin membranes and PLGA overcoat, ensuring a leak-proof perfusable vascular network formation. The middle cell-laden fibrin membrane exhibited strong barrier functions to the fluid flow similar to native blood capillaries (Table 1). The induced ECs are assembled into capillary-like structures spontaneously in the middle layer of the spindle construct after certain time of in vitro culture. The inherent ability of the induced ECs to arrange into capillary-like structures increased the integrity of the hierarchical vascular networks.

A major advantage of this strategy in organ manufacturing lies in the fact that it allows immediate perfusion of the large construct after the manufacture process. The integrity of the spindle construct remained for more than several months in the belly of rats [36]. Multiple heterogeneous types of cells grow, proliferate and/or differentiate simultaneously in the construct mimicking their native counterparts in micro- or macroenvironments [38]. Water, gases, growth factors, nutrients and other bioactive agents diffuse in a complete controlled manner throughout the 3D construct. The diffusion distance is at least a few hundred micrometers before being consumed in the fibrin membranes. Cell activities, such as hepatocyte secretion and ADSC proliferation capacities, remain at a high level for over 9 days of in vitro culture. ADSCs successfully transform into ECs and hepatocytes corresponding to the growth factor engagements and heterogeneous cell co-cultures (Figure 3, Figure 4, Figure 5, Figure 6 and Figure 7). In the future, glucose solution or culture medium may be used to measure the permeability of the constructs to better mimic the blood flow in the vascular networks.

Emphatically, after the fibrin membrane is formed and the internal mold is removed from the semi-construct, a branched cylindrical channel network is created that could be readily available for blood or fluid perfusion. The diameters of the branched vascular vessels ranged from hundreds of microns to several millimeters. The fabrication procedures have no detected adverse effects on the cell growth, proliferation, and differentiation capabilities in the spindle 3D constructs. This is compatible with a wide variety of cell types. The morphologies of the endothelial-like cells (i.e., the differentiated ADSCs) located on the surfaces of the branched vascular channels differ distinctly responding to the initial fibrinogen concentrations.

The usage of the natural polymer fibrinogen, the main structural protein in blood and thrombus, has several advantages over other biomaterials in loading and immobilizing the heterogeneous types of cells in the mechanically strong enough PLGA support constructs for bioartificial organ manufacturing. Firstly, the inherent biocompatibility of the natural fibrin, acting as the ECMs for ADSCs and hepatocytes, is well preserved throughout the manufacturing processes. In our previous reports, the dense fibrin matrices, with concentrations ranging from 1 to 4% (g/mL), were mechanically strong enough to retain the structural integrity of the cell-laden constructs [37,38,39]. The present study results are consistent with our former reports. Secondly, the micropores in the fibrin membranes are big enough for water, gas, growth factor, and nutrient passing. This leads to the formation of the hierarchical vascular network with a confluent EC layer behaving similar to that of a native vascular tree. The capacity of confluent EC layer formation depends largely on the initial fibrinogen concentration and ADSC density. The concentrations of the initial fibrinogen in the present study can be much lower than those in our previous 3D bioprinting technologies. ADSCs embedded in the fibrin hydrogels can either form large spheroids over time if there is no growth factor interference, or be induced into confluent endothelial cell layer with a cocktail growth factor engagement in the culture medium. Thirdly, there are many different means of stem cell engagement. The growth factors can be either incorporated directly in the cytocompatible fibrinogen solution before cell assembly or delivered through a culture medium after the manufacturing processes [55,56,57,58]. Furthermore, ADSCs embedded in the fibrin membranes can be induced into hepatocytes by controlling the release of hepatic growth factors in the neighboring hepatocyte-laden fibrin membrane (Figure 7).

The spindle endothelialized liver tissue combines the advantages of both the natural and synthetic polymers, with excellent cell compatibility and desired mechanical properties. Several distinctive achievements have been achieved using this technology: (i) a predefined multi-branched vascular network with adequate mechanical strength required for in vivo implantation; (ii) a precise control over the internal/external architectures of the vascularized liver tissues for multiple cell survivals and hepatic functionalities; (iii) an ingenious capillary-like network in between the multi-branched arteriole- and venule-like structures to avoid “short circuits” of the perfusable blood and/or fluid; (iv) a low concentration of fibrinogen can be used as the ECMs for multiple cellular incorporation and heterogeneous tissue generation; (v) a subtly stem cell engagement approach to ensure new tissue formation and subsequently complex organ maturation.

These results indicate that the presented technology represents significant progress in large vascularized organ—such as the liver—manufacturing. Its initial application in endothelialized liver tissue engineering has opened a new landscape to researchers and holds the potential to be widely used in the future complex organ manufacturing areas. It also provides an excellent 3D biomimetic model for studying the mutual cell-signal, cell-cell and cell-matrix interactions, stem cell differentiation conditions, heterogeneous cellular influences, multiple tissue in-growth mechanisms and target organ maturation processes. The ability to form such a functional multi-branched vascular network could therefore greatly expand the application range of the organ manufacturing products, such as high-throughput drug screening, failure organ restoration, ischemic necrosis treatment, and malignant tumor therapy.

## 5. Conclusions

The unique ACMS technology allows independent control of an implantable endothelialized liver tissue with special geometrical structures, mechanical properties and heterogeneous cell fates in a large predesigned spindle construct. Two different cell types, including ADSCs and hepatocytes, are arranged into specific locations in the predesigned spindle construct through the Weissenberg effect of a cell-laden non-Newtonian fibrinogen fluid. During the earlier fabrication stages, ADSCs and hepatocytes are irreversibly encapsulated in the fibrin membranes after the fibrinogen molecules polymerized. Cell activities, such as hepatocyte secretion and ADSC proliferation, are retained. ADSCs encapsulated in the fibrin membrane have been induced into both ECs and hepatocytes with growth factor engagements and cell cocultures. With the cocktail endothelial growth factor engagement, ADSCs quickly aligned into well-organized structures either along the branched channels or in the middle connection layer. The fibrinogen concentrations play an important role in the regulation of the differentiated endothelium phenotypes. Hepatocytes inside the fibrin membrane are capable of carrying out liver-specific functions, such as albumin synthesis. A complete vascular network, comprising a central artery-like, a central vein-like, 7 branched arteriole-like, 7 branched venule-like, and abundant capillary-like structures, was produced, allowing the combination of the good mechanical properties of the synthetic polymer PLGA and the excellent cyto-compatibilities of the natural polymer fibrin. The endothelialized vascular network can be connected to the host vasculature directly to fulfill the required commitments of nutrient, gas and waste exchange for the incorporated cells. This unique ACMS technology holds great promise for the design and manufacture of elaborate important visceral organs, such as the liver, heart, and kidney, in the near future. It therefore presents a significant step in bioartificial organ manufacturing areas and holds the potential to be widely used in the related fields, such as cell transplantation, drug screening, pathological analyses, tissue engineering and organ restoration.

## Figures and Tables

**Table 1:** Permeation times of the HCI solution in the three groups of spindle constructs.

Group	Permeation Time (s)
A	61.4 ± 8.1
B	57.1 ± 8.3
C	47.8 ± 7.5
